# Nanoporous Structure Formation in GaSb, InSb, and Ge by Ion Beam Irradiation under Controlled Point Defect Creation Conditions

**DOI:** 10.3390/nano7070180

**Published:** 2017-07-11

**Authors:** Yusuke Yanagida, Tomoya Oishi, Takashi Miyaji, Chiaki Watanabe, Noriko Nitta

**Affiliations:** 1 School of Environmental Science and Technology, Kochi University of Technology, Tosayamada, Kami, Kochi 782-8502, Japan; willowhellow@gmail.com (Y.Y.); 205006q@gs.kochi-tech.ac.jp (T.O.); specialweek.25.25.25@gmail.com (T.M.); wtnb.1415@gmail.com (C.W.); 2 Center for Nanotechnology, Research Institute, Kochi University of Technology, Tosayamada, Kami, Kochi 782-8502, Japan

**Keywords:** nanoporous structure, GaSb, InSb, Ge, ion beam irradiation, point defect, interstitial, vacancy, surface modification, FIB

## Abstract

Ion beam irradiation-induced nanoporous structure formation was investigated on GaSb, InSb, and Ge surfaces via controlled point defect creation using a focused ion beam (FIB). ‎This paper compares the nanoporous structure formation under the same extent of point defect creation while changing the accelerating voltage and ion dose. Although the same number of point defects were created in each case, different structures were formed on the different surfaces. The depth direction density of the point defects was an important factor in this trend. The number of point defects required for nanoporous structure formation was 4 × 10^22^ vacancies/m^2^ at a depth of 18 nm under the surface, based on a comparison of similar nanoporous structure features in GaSb. The nanoporous structure formation by ion beam irradiation on GaSb, InSb, and Ge surfaces was controlled by the number and areal distribution of the created point defects.

## 1. Introduction

Nanoporous structures on semiconductor surfaces have important application potential for electronic and photonic devices. Ion beam irradiation-induced nanoporous structure formation on gallium antimonide (GaSb) [1,2,3,4,5,6,7,8,9,10,11,12], indium antimonide (InSb) [1,13,14,15,16,17,18,19,20], and germanium (Ge) [21,22,23,24,25,26,27,28,29] surfaces have been studied previously, with D. Kleitman and H. J. Yearian being the first to report this phenomenon using deuteron irradiation of GaSb and InSb in 1957 [1]. Such nanoporous structure formation by ion beam irradiation has only been observed on GaSb, InSb, and Ge surfaces; Si [30], GaAs [31,32], and InP [33] surfaces were not formed in this way. Layer damage and amorphous structure formation were only observed as a result of high dose irradiation. Recently, similar nanoporous structure formation behavior was also observed in Si_1−*x*_Ge*_x_* [34,35] and GaAs_1−*x*_Sb*_x_* [36] irradiated alloys; this formation behavior on Si_1−*x*_Ge*_x_* and GaAs_1−*x*_Sb*_x_* was likely influenced by Ge and GaSb, respectively. This nanoporous structure formation mechanism has been previously labeled as the migration of ion beam irradiation-induced point defects (Frenkel pair; interstitial and vacancy) [5]. Many point defects are generated near the surface by collisions cascade from ion beam irradiation. Small voids or elevations are formed in the early stage of irradiation due to numerous interstitials and vacancies. The surface roughness increases due to the migration of these interstitials and vacancies as a result of nanoporous structure formation on the surface. The amount of point defects necessary to impact structure formation is also questionable in this mechanism. The dependence on ion dose on the nanoporous structure size has been reported in numerous prior studies. However, the relationship between the number of point defects and the nanoporous structure formation has not yet been examined in detail.

In this paper, ion beam irradiation-induced nanoporous structure formation was investigated on GaSb, InSb, and Ge surfaces by controlled point defect creation using a focused ion beam (FIB). An accelerator was used with the FIB to allow the examination of a number of different ion beam conditions. The nanoporous structure formation mechanism here is predominantly influenced by point defect behavior. If the same number of point defects is created, the same nanoporous structure formation behavior is expected. This paper compares nanoporous structure formation on these surfaces under different accelerating voltages and ion doses, while keeping the number of point defects constant. The point defects were calculated using stopping and range of ions in matter (SRIM) simulations [37]. The aim of this study is to develop ion beam conditions for the synthesis of nanoporous structures in order to determine the influence of the number of point defects created.

## 2. Experimental Procedure

FIB ion beam irradiation was conducted using Ga^+^ with an FEI Quanta 3D 200i at room temperature. Single crystals of GaSb, InSb, and Ge (001) as mirror-polished wafers were used. The accelerating voltages were 2, 5, 8, 16, and 30 kV, at a chamber vacuum of 4 × 10^−4^ Pa. The Ga ion irradiation utilized an image scanning mode, in which Ga was irradiated in a 512 × 441 dot array over a 12.5 μm × 10.8 μm area of the surface in a single scan. The scanning dose was 5 × 10^18^ ions/m^2^ for each scan. 2–84 to eighty-four scans were performed for GaSb and InSb, while 10–240 scans were used for Ge. The total beam dose was 5 × 10^18^–1.4 × 10^21^ ions/m^2^. Structural changes resulting from ion beam irradiation were observed by field-emission scanning electron microscopy (FE-SEM; JEOL JSM-7401F). The accelerating voltage was 5 kV.

## 3. Results and Discussion

**Figure 1:**
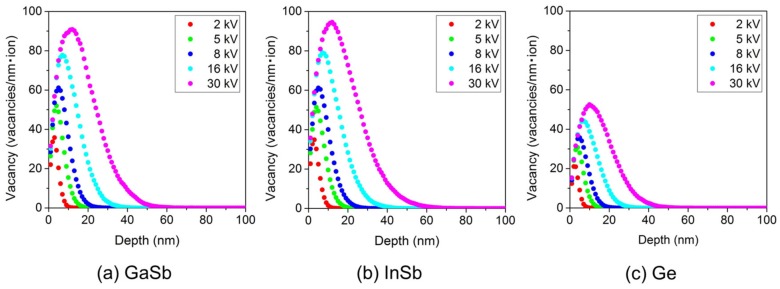
Vacancy distributions of (**a**) GaSb, (**b**) InSb, and (**c**) Ge irradiated with a Ga ion beam as a function of distance from the surface, calculated by SRIM simulations. Accelerating voltages were 2, 5, 8, 16, and 30 kV.

Figure 1 shows the vacancy distributions of (a) GaSb, (b) InSb, and (c) Ge irradiated with a Ga ion beam as functions of distance from the surface, calculated using SRIM simulations [37]. Table 1 summarizes the projected range and vacancies per ion in (a) GaSb, (b) InSb, and (c) Ge, as also calculated by SRIM simulations. SRIM is a Monte Carlo simulation of ion beam collisions in solids. The number of calculated Ga ions was 10,000. We adopted displacement threshold energy values obtained by Thommen (6.2 eV for Ga and 7.5 eV for Sb) [38], Bauerlein (5.8 eV for In and 6.8 eV for Sb) [39], and H. H. Andersen and J. F. Ziegler (15 eV for Ge) [40]. The projected depth and number of vacancies per ion increased with the increasing accelerating voltage in all calculations. The vacancy distribution tendency was nearly identical in GaSb and InSb, while Ge featured half as many vacancies as those. The irradiation doses for the below experiments were determined by these point defect numbers, resulting in nearly identical numbers of vacancies on these irradiated surfaces. Here, we should consider the influence of the implanted Ga ions. The Ga ion concentration was calculated in GaSb unit area. The unit of irradiated region in GaSb 100% was equal to 0.7% Ga ions (5 × 10^18^ ions/m^2^ scan). Therefore, the influence of the implanted Ga ions is clear in this experiment.

**Figure 2:**
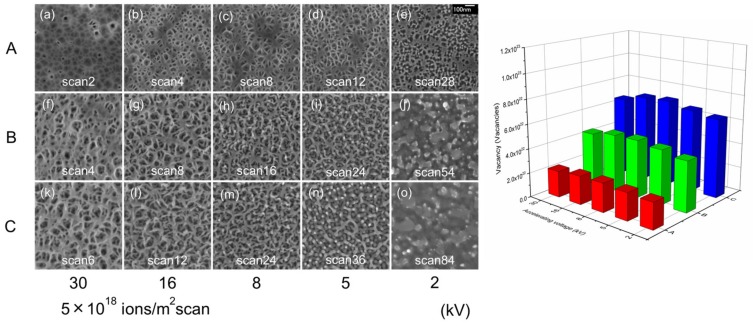
Surface SEM images of GaSb irradiated with a Ga^+^ ion beam at room temperature (**left**). The accelerating voltages used were 2, 5, 8, 16, and 30 kV at a scanning dose of 5 × 10^18^ ions/m^2^ per scan. The total number of vacancies created on GaSb was estimated using the ion dose (ions/m^2^) multiplied by the vacancy count (/ion) as calculated by SRIM simulations, as a function of accelerating voltage (**right**).

Figure 2 shows surface SEM images of GaSb irradiated with a Ga^+^ ion beam at room temperature (left), at accelerating voltages of 2, 5, 8, 16, and 30 kV. The total number of vacancies created on the GaSb surface was estimated using the ion dose (ions/m^2^) multiplied by the vacancies (/ion) from the SRIM simulation as a function of accelerating voltage (right). Column A shows an average number of vacancies of 2.3 × 10^22^ vacancy/m^2^, column B shows 4.5 × 10^22^ vacancy/m^2^, and column C shows 6.8 × 10^22^ vacancy/m^2^; very similar numbers of vacancies are present in all cases. The nanoporous structure formation was examined under different accelerating voltages and ion dose levels, with a fixed number of point defects. Despite this fixed point defect count, the same structure was not formed on all surfaces, and the nanoporous structure features are different in each column. Voids were formed under the surfaces in Figure 2a, while cavities were observed on the surfaces in Figure 2b–e. In comparing columns A, B, and C, the structure size increases with increasing ion dose. Decreasing the accelerating voltage causes the structure to change from a thin walled setup to more uneven, rugged features. The largest structure size was observed at an acceleration voltage of 16 kV, while surface roughness is observed in Figure 2j,o, resulting from a lower acceleration voltage of 2 kV.

**Figure 3:**
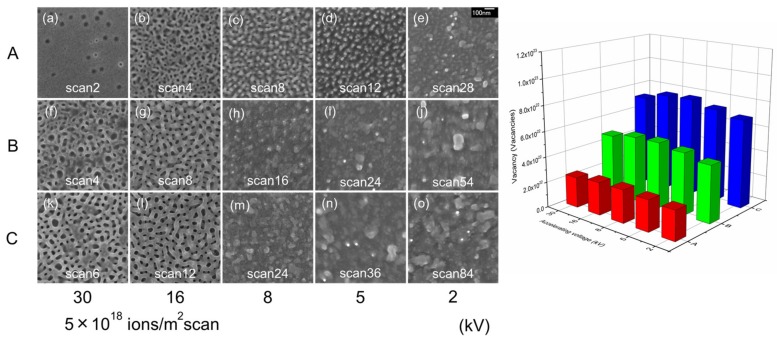
Surface SEM images of InSb irradiated with a Ga^+^ ion beam at room temperature (**left**). The accelerating voltages used were 2, 5, 8, 16, and 30 kV at a scanning dose of 5 × 10^18^ ions/m^2^ per scan. The total number of vacancies created on InSb was estimated using the ion dose (ions/m^2^) multiplied by the vacancy count (/ion) as calculated by SRIM simulations, as a function of accelerating voltage (**right**).

Figure 3 shows surface SEM images of InSb irradiated with a Ga^+^ ion beam at room temperature (left), at accelerating voltages of 2, 5, 8, 16, and 30 kV and a scanning dose of 5 × 10^18^ ions/m^2^ per scan. The total number of vacancies created on the InSb were estimated using the ion dose (ions/m^2^) multiplied by the vacancy count (/ion) calculated using SRIM simulations as a function of accelerating voltage (right). Column A indicates an average vacancy count of 2.5 × 10^22^ vacancy/m^2^, column B shows a value of 4.9 × 10^22^ vacancy/m^2^, and for column C this number is 7.4 × 10^22^ vacancy/m^2^. As in the case of GaSb, the same structure was not formed on the InSb surface despite the same number of point defects being present. Compared to GaSb, more spherical structures were present in InSb, in terms of both void and elevation structures. These spherical structures likely formed due to the decreased surface energy of this system. Under low accelerating voltage irradiation, rugged structural features emerged in a similar manner as GaSb. The mechanism of InSb nanoporous structure formation has been previously reported as a combination of sputtering and re-deposition [17,18]. Table 2 shows sputtering yield (atoms/ion) calculated by SRIM simulations [37] in (a) GaSb, (b) InSb, and (c) Ge. The sputtering yield in InSb was higher than that in GaSb. This sputtering is more effective for nanoporous structure formation compared to point defect migration in InSb.

The nanoporous structure was not observed on GaSb in Figure 2j,o, and on InSb in Figure 3e,h–j,m–o at low accelerating voltages. It was considered that the influence of sputtering was effective at low accelerating voltages. In spite of the fact that the sputtering yield was low with decreasing accelerating voltage in Table 2, the nanoporous structure was not formed. This sputtering is also more effective for nanoporous structure formation compared to point defect migration at low accelerating voltages. It was considered that the number of point defects was few at accelerating voltages.

**Figure 4:**
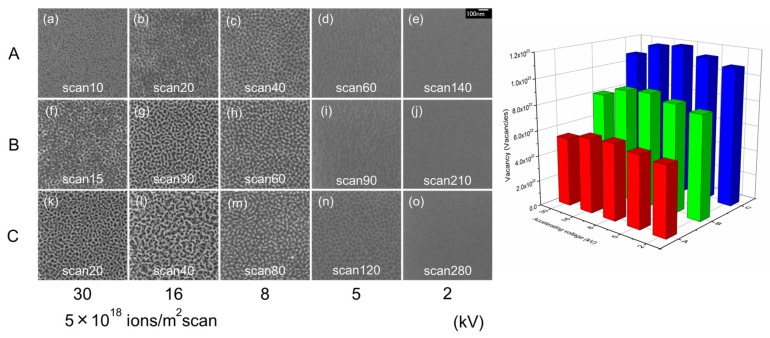
Surface SEM images of Ge irradiated with a Ga^+^ ion beam at room temperature (**left**). The accelerating voltages used were 2, 5, 8, 16, and 30 kV at a scanning dose of 5 × 10^18^ ions/m^2^ per scan. The total number of vacancies created on Ge was estimated using the ion dose (ions/m^2^) multiplied by the vacancy count (/ion) as calculated by SRIM simulations, as a function of accelerating voltage (**right**).

Figure 4 shows surface SEM images of Ge irradiated with a Ga^+^ ion beam at room temperature (left), at accelerating voltages of 2, 5, 8, 16, and 30 kV and a scanning dose of 5 × 10^18^ ions/m^2^ per scan. The total number of vacancies created on the Ge surface was estimated using the ion dose (ions/m^2^) multiplied by the vacancy count (/ion), as calculated by SRIM simulations [37], as a function of accelerating voltage (right). Column A indicates an average vacancy count of 5.7 × 10^22^ vacancy/m^2^, column B denotes this value as 8.5 × 10^22^ vacancy/m^2^, and column C is 1.1 × 10^23^ vacancy/m^2^. The number of vacancies formed in Ge, based on our SRIM simulations (Table 1), was lower than in both GaSb and InSb. Therefore, the ion dose reaching the surface during Ge irradiation is approximately double what was present for GaSb and InSb. Smaller and finer structures were formed on the Ge surface compared to these other surface types, despite the greater ion dose experienced by Ge. This can be explained by the different formation mechanisms between Ge, GaSb, and InSb. In the case of Ge, an amorphous structure first forms on the Ge surface, from which the final nanoporous structure grows [25]. A high ion dose is thus needed for this initial amorphization stage.

**Figure 5:**
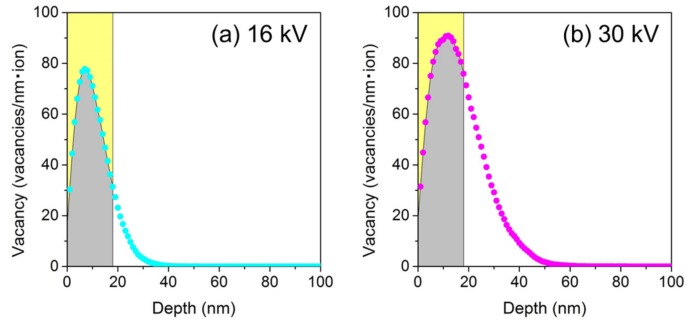
Vacancy distributions of GaSb irradiated with a Ga ion beam as a function of distance from the surface, calculated using SRIM simulations [37] (re-arranged in Figure 1a). Accelerating voltages were (**a**) 16 kV and (**b**) 30 kV. The gray hatched regions indicate the region 18 nm below the surface.

The nanoporous structure size is expected to decrease with the decreasing accelerating voltages. However, the largest structure features were observed 16 kV irradiation (Figure 2, Figure 3 and Figure 4). The reason for this behavior is based on the density of the created point defects. As shown in Figure 2g,k, similar GaSb structures were formed in spite of the different number of point defects present in both cases. The ion beam conditions and number of point defects are compared for these samples in Table 3. The accelerating voltage was 16 kV; compared to an accelerating voltage of 30 kV, the ion dose was 1.3 times higher and the number of point defects was 0.7 that of the latter voltage. The number of point defects was low in the case of 16 kV irradiation, but large-scale structures were formed. To compare the distribution of point defects in the depth direction, Figure 5 shows the vacancy distributions of GaSb irradiated with a Ga ion beam as a function of distance from the surface (re-arranged from Figure 1a). The accelerating voltages were (a) 16 kV and (b) 30 kV. The gray hatched regions are 18 nm below the surface. The integrated number of vacancies at this depth was 1363 at 16 kV and 1034 at 30 kV. The number of point defects was estimated at this depth based on the ion dose (ions/m^2^) and vacancies present (/ion). It is expected that these vacancies require the same formation features as the nanoporous structures. The number of point defects required for nanoporous structure formation was 4 × 10^22^ vacancies/m^2^ at a depth of 18 nm from the surface in GaSb.

These evaluated vacancy counts have been validated by previous experiments. In 800 kV Cu^+^ [41] and 270 kV C_60_^2+^ [42] fullerene ion beam irradiation, a vacancy count of 4 × 10^22^ vacancies/m^2^ appeared at this same depth from the surface in GaSb. Nanoporous structures were also formed on the surfaces in these conditions, despite the different ion species present. This indicates that, while the ion beam conditions such as ion dose, accelerating voltage, and ion species differed, the number of vacancies and their distribution was much more important. Controlled point defect creation is useful for nanoporous structure formation using ion beam irradiation on GaSb, InSb, and Ge surfaces.

## 4. Conclusions

Ion beam irradiation with the same number of created point defects led to different types of nanoporous structure formation on GaSb, InSb, and Ge surfaces. The depth direction density of the point defect distribution was an important factor for nanoporous structure formation. The required number of created point defects for nanoporous structure formation was 4 × 10^22^ vacancies/m^2^ at a depth of 18 nm from the surface in the case of GaSb. Nanoporous structure formation by ion beam irradiation on GaSb, InSb, and Ge surfaces can be controlled based on the number and distribution of point defects, which can serve as an index for nanoporous structure formation in general.

## Figures and Tables

**Table 1:** Projected range and vacancy per ion calculated by SRIM simulations in (**a**) GaSb, (**b**) InSb, and (**c**) Ge.

**(a) GaSb**					
**Acc. Vol. (kV)**	**2**	**5**	**8**	**16**	**30**
Ion range (nm)	3.6	6.1	8.2	12.9	20.2
Vacancy (/ion)	154	374	593	1169	2156
**(b) InSb**					
**Acc. Vol. (kV)**	**2**	**5**	**8**	**16**	**30**
Ion range (nm)	4.0	6.8	9.0	13.9	21.9
Vacancy (/ion)	166	408	647	1281	2382
**(c) Ge**					
**Acc. Vol. (kV)**	**2**	**5**	**8**	**16**	**30**
Ion range (nm)	3.2	5.5	7.3	11.7	18.3
Vacancy (/ion)	77	189	298	585	1076

**Table 2:** Sputtering yield (atoms/ion) calculated by SRIM simulations [37] in (**a**) GaSb, (**b**) InSb, and (**c**) Ge.

**(a) GaSb**					
**Acc. Vol. (kV)**	**2**	**5**	**8**	**16**	**30**
Total	3.553	4.939	5.671	6.367	6.928
III element	1.80	2.48	2.87	3.24	3.52
V element	1.75	2.46	2.80	3.13	3.41
**(b) InSb**					
**Acc. Vol. (kV)**	**2**	**5**	**8**	**16**	**30**
Total	4.142	5.822	6.660	7.975	8.537
III element	2.18	3.06	3.50	4.19	4.47
V element	1.96	2.77	3.16	3.78	4.07
**(c) Ge**					
**Acc. Vol. (kV)**	**2**	**5**	**8**	**16**	**30**
Total	2.965	4.215	4.810	5.766	4.801

**Table 3:** Comparison of ion beam conditions and point defects calculated by SRIM simulations [37] on similar structure formation of GaSb in Figure 2g,k.

Acc. Vol. (kV)	Scan	Scan Dose (ions/m2 scan)	Total Dose (ions/m2)	Vacancy (/ion)	Total Vacancy (/m2)	Vacancy (/ion) under 18 nm from the Surface	Total Vacancy (/m2) under 18 nm from the Surface
16	8	5 × 10^18^	4 × 10^19^	1169	4.7 × 10^22^	1363	4.1 × 10^22^
30	6	5 × 10^18^	3 × 10^19^	2156	6.5 × 10^22^	1034	4.1 × 10^22^
